# Scalable preprocessing of high volume environmental acoustic data for bioacoustic monitoring

**DOI:** 10.1371/journal.pone.0201542

**Published:** 2018-08-03

**Authors:** Alexander Brown, Saurabh Garg, James Montgomery

**Affiliations:** School of Technology, Environments and Design, University of Tasmania, Hobart, Tasmania, Australia; US Department of Agriculture, UNITED STATES

## Abstract

In this work, we examine the problem of efficiently preprocessing and denoising high volume environmental acoustic data, which is a necessary step in many bird monitoring tasks. Preprocessing is typically made up of multiple steps which are considered separately from each other. These are often resource intensive, particularly because the volume of data involved is high. We focus on addressing two challenges within this problem: how to combine existing preprocessing tasks while maximising the effectiveness of each step, and how to process this pipeline quickly and efficiently, so that it can be used to process high volumes of acoustic data. We describe a distributed system designed specifically for this problem, utilising a master-slave model with data parallelisation. By investigating the impact of individual preprocessing tasks on each other, and their execution times, we determine an efficient and accurate order for preprocessing tasks within the distributed system. We find that, using a single core, our pipeline executes 1.40 times faster compared to manually executing all preprocessing tasks. We then apply our pipeline in the distributed system and evaluate its performance. We find that our system is capable of preprocessing bird acoustic recordings at a rate of 174.8 seconds of audio per second of real time with 32 cores over 8 virtual machines, which is 21.76 times faster than a serial process.

## Introduction

Currently, many of the world’s ecosystems are vulnerable because of the impact of humans, though the means of, among other things, deforestation [1] and climate change [2]. As such, it has become critically important to monitor ecosystems, in order to derive conservation strategies to reduce human impact on the environment.

In order to adequately monitor the Earth’s ecosystems, analyses need to be carried out over large areas over long durations. As such, traditional approaches, such as having experts in locations of interests actively observing ecosystems are prohibitively expensive and infeasible [3].

Because of their ability to perform processes on larger scales at relatively low cost, it is becoming increasingly popular to utilise ecoacoustics approaches to efficiently monitor ecosystems using microphones to record sounds of the environment [4]. From there, researchers can either manually listen to recordings, or utilise computer algorithms to analyse the recordings. This can reveal important information about ecosystems, which can lead to productive decisions regarding conservation. In particular, ecoacoustics has been used frequently to study the impact of the biodiversity of ecosystems due to climate change [5].

Almost all bioacoustic analyses require audio to be preprocessed to get it into a form suitable for analysis. This may include data compression techniques to speed up processing such as removing unnecessary audio channels [6] and downsampling [7]. It can also include improving the quality of audio by reducing noise interference, which is a key challenge for many bioacoustic studies because noise can mask vocalisations of interest [3]. Many current bird classification techniques are evaluated using clean recordings (e.g. [6, 8, 9]), but these fail to confront this significant challenge, and might not work effectively in real world monitoring scenarios, which often rely on huge amounts of unsupervised environmental recording data (e.g. [10–12]).

Noise can be considered to be any sound that is not produced by a target organism. It is of great importance to remove these noises so that further processing (e.g. bird identification) can focus on the parts of a recording containing bird sound without interference. Many approaches already exist for detecting and removing noise from multiple sources [13–17].

Current bioacoustic preprocessing approaches typically do not use multiple processes to remove noise from multiple sources and, as such, do not consider the order in which processes should be applied in any depth. Accordingly, current processing is potentially slower and less accurate than it could be. This presents a key challenge: to determine which order of processing tasks will result in the most efficient and effective pipeline possible. An investigation of the ordering of preprocessing tasks could improve current processing for a variety of bioacoustic applications, such as biodiversity appraisal [18] and species classification processes [19].

Additionally, many bioacoustic preprocessing approaches are applied individually in a manual or semi-automated way. However, such approaches are not well suited to large scale studies because of the time required to process recordings [3, 9, 20]. Recorders are being deployed in larger numbers across different natural environments, and so are collecting bioacoustic data at high volumes, sometimes on the order of hundreds of gigabytes per day [21]. Processing environmental recordings on this scale is not viable in terms of both cost and time with existing methods [22].

We address the key challenge of how to improve the computational speeds of preprocessing tasks in a cost efficient manner. We propose to combine preprocessing tasks into a pipeline and distribute this pipeline among several processors, using a master-slave system to achieve this. Determining how to best distribute processing needs to be investigated. Ideally, the system should be linearly scalable. This means that improvements in execution time (i.e. in terms of ratios) should be linearly proportional to the number of processors used.

A potential option for distributing processing tasks is to use an off-the-shelf system such as Hadoop [23] or Spark [24], although these do not give low-level control over data in order to maximise efficiency and introduce significant overhead. A previous attempt to utilise Hadoop and Spark for some preprocessing steps (such as splitting bioacoustic audio files and generating spectrograms) by Thudumu et al. [25] did not achieve linear scalability, and only performs a simple process.

Combining all processes together is not a simple matter of performing one process after another, or performing any process in random order, because each process might have an impact on the effectiveness of subsequent processes and the overall execution time of the processing pipeline. For example, a stationary noise filter might have an effect on how accurately rain can be detected and filtered. If heavy rain can be detected accurately without using a stationary noise filter, then the stationary noise filter does not need to be applied on data known to contain rain, under the assumption that rain interference irreparably damages any bird signal in the audio. Complicating matters further, rain might be detected more accurately after stationary noise reduction, but overall processing will be slower, so there is a trade-off between accuracy and efficiency. As such, finding the order of execution for preprocessing filters is important and non-trivial to solve.

To address these challenges, we investigate several factors which could influence the efficiency of our proposed preprocessing pipeline; most significantly, the order in which preprocessing tasks are executed. This involves thoroughly investigating the execution times of individual tasks, and the effects of split length on the accuracy of several filters. We also investigate several parameters within our proposed distributed system that influence the data processing efficiency of the system.

To summarise, the primary contributions of this paper are:

- Design of a pipeline combining multiple processes to produce a system for preprocessing environmental recordings. We perform a thorough investigation to determine the best order of processing tasks for effective and efficient preprocessing, including efficiency and accuracy trade-offs.
- Design of a highly scalable distributed system built specifically for the preprocessing pipeline, utilising data parallelism to increase the speed of processing while ensuring all processors are highly utilised and maintaining low overhead.

We describe the distributed computing system used to execute the preprocessing pipeline, then determine the best order of execution for this pipeline. The performance of the pipeline, when run on the distributed system, is then evaluated.

## Proposed distributed system for preprocessing

This section describes the proposed system for distributing work amongst multiple machines. The processing pipeline is subsequently derived and distributed by this system.

### Master-slave model

Our approach utilises a master-slave architecture with file parallelisation to progress through the processing pipeline. Files are processed through the pipeline on one slave each. This approach is selected, as opposed to distributing work on a per-process basis, because workload can be evenly distributed among slaves by splitting files into small chunks.

The master first splits each file. Based on our investigation of individual processing tasks, we determine if the master process should also perform other processes. Upon finishing splitting and any other initial processing, the master adds files into a queue. The master and slaves then communicate with each other when they are ready to send and receive files. Slaves complete what is left of the processing pipeline before sending the output back to the master.

Slaves will store chunks that have completed processing in a queue and send the results of all finished processes at constant time intervals. This is preferable to sending one file at a time because the amount of communication between the master and slave threads is lower.

The master tracks which files have been sent to each slave, and which have completed processing, such that it can re-send files to different slaves if a slave disconnects or crashes.

### Slave parallelisation

Parallelisation is performed both between multiple machines and between multicore processors. To parallelise work within a single machine, a central thread handles communication between the master and the slave, acting similarly to a secondary master (with threads being slaves). Files given to the slave from the master are added to a queue of files pending processing, which is managed by the central thread. The queue is set to a fixed size, such that if the queue falls below this size, the slave will request more files from the master. Processing threads then remove files from the queue and process them through the denoising pipeline. Upon completing processing, files will enter another queue, from which they will be sent back to the master process. If the file is deleted at some point during denoising (e.g. because it contains heavy rain), the file name is sent back to the master to indicate that it has been processed.

### Distribution of work

This system needs to perform multiple preprocessing tasks so that bird acoustic data can be effectively analysed. There are several factors that need to be investigated in order to efficiently distribute work amongst multiple machines. The first of these is to determine which order work should be executed. This is significant because some processes affect the accuracy of other processes. Additionally, some processes might make others redundant for some of the data.

Another challenge involves determining which length of audio each slave should process at a time. This affects the accuracy of some processes. Additionally, there is a trade-off between minimising the amount of communication between the master and the slaves, which is achieved by sending more work to do at once, and ensuring workloads are processed evenly, which is easier to do if less work is done at once.

## Processing pipeline

### Investigation of the best sequencing of tasks

We now focus on determining the best order of execution for an efficient preprocessing pipeline for use in our distributed system. The pipeline is derived by first evaluating execution times for each process, and how this varies with the lengths of audio chunks processed at a time. We then evaluate the accuracy of noise detection processes before and after applying a stationary noise filter (Minimum Mean Square Error Short Time Spectral Amplitude estimator, or MMSE STSA [14]), and finally test to see if detection approaches have a dependency on split length.

### Experimental design

Three experiments are conducted to assist in the development of the preprocessing pipeline. The first experiment looks at the computation times for each processing step, and how these vary depending on the size of data they are processing at once (called file split size/length). This experiment identifies fast and slow processes. Faster processes are placed earlier in the pipeline where possible if they can result in later, slower processes being skipped for some data (i.e. due to the deletion of audio). This experiment can also help to identify which split lengths result in faster execution for each process, which can be used to improve their execution time.

For bird detection problems, ideally files should not be split such that individual bird calls are split across two files, but this is not a problem for noise reduction, because the noise sources being examined occur over longer time periods. Files might need to be merged for bird detection, although this is not a consideration taken in this preprocessing pipeline. An overlap between splits could be added for bird detection problems.

The second experiment examines the effect of the MMSE STSA filter, which alters audio files in a significant way and hence affects detection processes. As such, we test the accuracy of detection approaches before and after applying the filter.

The final experiment looks at whether detection accuracy is dependent on the length of chunks into which the audio is split. We take a random 30 minute sample of unsupervised recordings, manually classify rain and cicada choruses and compare this to the automatic classifiers. This can show if detectors work better on certain lengths. This is important in determining the processing order, because it is easier to split audio files than to join them together, because joining requires that consecutive chunks are processed on the same machine. This means that detection processes with longer split lengths should run earlier than those that do not.

#### Recording data

Environmental recordings for evaluating the system have been provided by the Samford Ecological Research Facility (SERF), based in the Queensland University of Technology (QUT). These recordings were taken over five days between 12 October 2010 and 16 October 2010, over four sensors, for a total of 20 days of audio to process. In practice, four days of recordings are used in testing. Recordings from this group have been used in several studies [16, 20, 21]. While these recordings are of high quality, they do contain significant levels of background noise, large variations in the loudness of bird sounds (ranging from very clear to barely audible)m and noise interference from many sources including rain and cicadas, which makes the sample well suited for this study.

### Pipeline processes

The preprocessing stage consists of the following tasks:

- **Splitting**: Audio is split into smaller chunks which allows for work to be distributed more easily. Additionally, long files are not viable for processing on their own because of high RAM requirements [21], and some classification tasks in the pipeline work better on shorter samples.
- **Downsampling**: Audio files have sample rates converted to 22.05 kHz to reduce their size. Bird sounds are normally below 11.025 kHz (the Nyquist frequency) [26], so signals of interest are normally not lost.
- **Converting to Mono**: Only one channel of audio is needed to detect significant audio signals, so this is used to further reduce the size of files.
- **High-Pass Filter (1 kHz)**: Birds typically do not emit sound below 1 kHz [26], so all data below this frequency is noise and hence is attenuated.
- **Sound Enhancement**: Stationary background noise is reduced. While there are several approaches that can achieve this [17, 27, 28] we use the Minimum Mean Square Error Short Time Spectral Amplitude estimator (MMSE STSA) filter [14], which was found in separate work [3] to be highly effective. Our previous work [29] showed that this was more effective and time efficient than alternatives.
- **Short-Time Fourier Transform**: Time-based information is transformed into frequency-based information. Several acoustic indices used in cicada and rain detection use frequency-based information, so this is only executed once, rather than for each acoustic index calculated, or for each process. The FFT implementation used here is from the Apache Commons Math library [30] and is described by Demmel [31]. A window size of 256 samples is used with Hamming windows with 50% overlap.
- **Heavy Rain Detection and Removal**: Heavy rain is detected by using rules derived from a C4.5 classifier [32] using acoustic indices. This approach is similar to Towsey et al. [16] and Ferroudj [15]. Spectral-based signal to noise ratio and power spectral density used by Bedoya et al. [13] were added to the acoustic indices used in the classifier. The classifier was trained on a separate sample of data and its rules then hard coded into our Java-based implementation prior to beginning the pipeline.
- **Cicada Detection**: Cicada choruses are detected using the same general approach as rain detection.
- **Cicada Removal**: Cicada choruses are removed using band-pass filters to eliminate audio from frequency ranges containing cicada choruses. These ranges are calculated by examining FFT coefficients. Although it is possible that this filter will remove some bird signal, the cicada chorus is usually significantly louder than bird signals. As such, we assume that any bird signal in the same frequency region as the cicada chorus could not be accurately analysed because of extra noise interference.

#### Per-step execution time

A test is conducted where each step is performed independently. Two hours of audio known to contain rain, cicada choruses and bird sounds is passed through the processing pipeline in sequence, using one processor. The split length is varied (from 5 to 30 seconds in 5-second increments) to observe its effects on processing time. Each test is completed five times for each split length, and the average and standard deviation of the computation times are taken.

**Fig 1:**
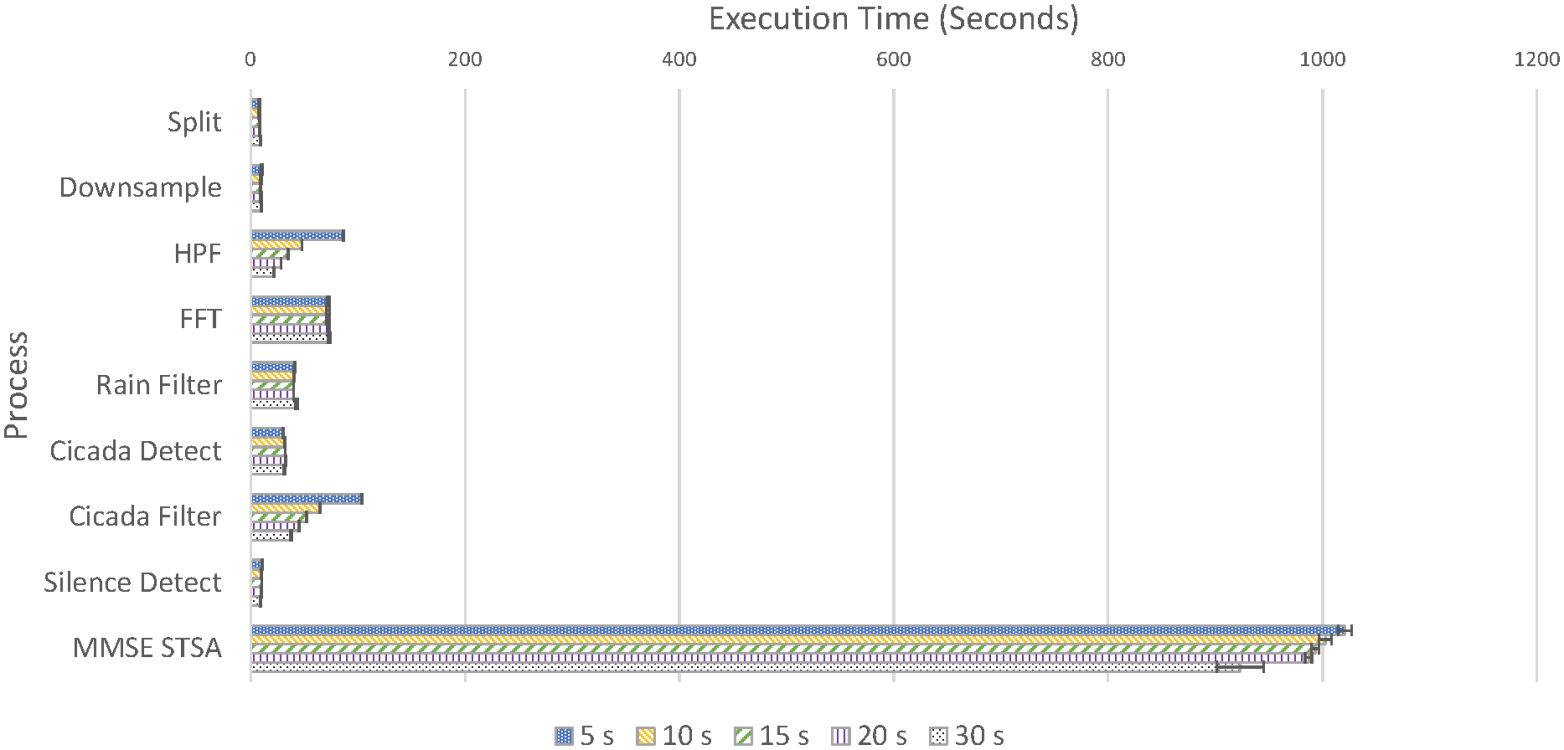
Computation times per process for different split lengths up to cicada detection. Error bars indicate standard deviation (FFT = Fast Fourier Transform, HPF = High-Pass Filter, MMSE STSA = Minimum Mean Square Error Short Time Spectral Amplitude filter).

Fig 1 and Table 1 shows the execution times for all processes for 2 hours (1.2 GB) of audio. Each process is applied to every file, although, once the pipeline is developed, not all processes are applied to every file, because some files containing rain may be removed early.

**Table 1:** Computation times for each processing step in relation to split lengths with standard deviations.

Processing Step	Split Length (seconds)				
	5	10	15	20	30
Splitting	7.85±0.42	7.95±0.49	8.13±0.51	9.24±0.42	8.87±0.42
Downsampling	10.18±0.42	9.59±0.68	9.30±0.30	9.29±0.52	9.57±0.19
High-pass Filter	86.63±0.13	47.79±0.17	34.8±0.18	28.2±0.11	21.67±0.09
Fast Fourier transform	2.39±1.01	47.79±1.44	71.90±1.36	73.15±0.56	73.21±0.95
Rain Filter	41.11±0.20	40.46±0.20	39.86±0.15	39.94±0.18	42.67±1.16
Cicada Detection	30.47±0.20	31.58±0.20	32.04±0.08	32.32±0.26	31.36±0.60
Cicada Filter	103.48±0.56	64.30±0.18	51.94±0.22	45.27±0.23	37.46±0.52
MMSE STSA	1020.57±6.49	1002.65±5.98	993.10±3.39	986.92±3.09	923.21±21.78

The figure shows two distinctive features. First is the large decrease in the execution time of the high-pass, cicada, and MMSE STSA filters filters when the split size is larger. The differences in high-pass and cicada execution times are likely due to the use of the non-native sound processing library Sound eXchange (SoX) [33]. This causes extra overhead with each call, and shorter split sizes require more calls to SoX. This is more of a problem for high-pass filtering than cicada filtering, as this is executed on every file, whereas cicada filtering only applies to parts of the recording where cicada choruses are detected, which, as determined by subsequent testing, is a small fraction of the total recording.

The second observation is that the MMSE STSA filter takes much longer than the other processing steps combined. As such, execution time can be significantly saved by removing audio before the MMSE STSA filter is applied.

**Fig 2:**
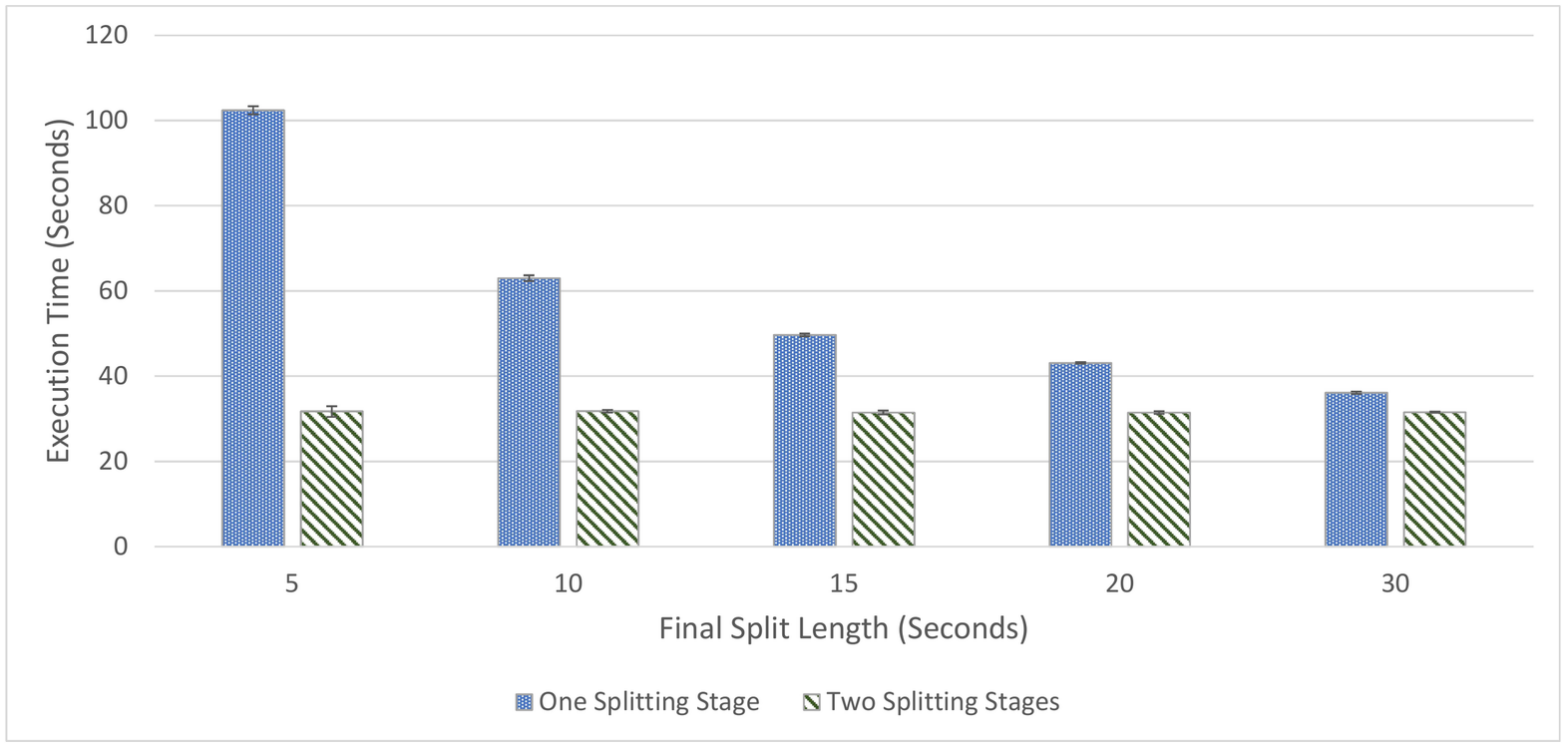
High-pass filtering computation times. High-pass filtering computation times comparison, between splitting to the final length, downsampling, and then high-pass filtering (one split) and splitting to 1-minute (2.5 MB) chunks first, downsampling and high-pass filtering, then splitting to the final length (two splits).

The trend in high pass filter execution time gives rise to a potential improvement. If clips are split into larger chunks first, downsampled and high-pass filtered, and then split into smaller chunks, execution time can be improved. Testing an approach that performs this shows an improvement in execution time, as shown in Fig 2. Here, audio is split into 1-minute (2.5 MB) long chunks, downsampled, high-pass filtered, and then split to the target split length. Two hours of audio is tested against two approaches, one that splits audio to the target length immediately, and one that split files into 1-minute long chunks first, and then splits again.

While it would be theoretically optimal to run a high-pass filter on whole audio files, rather than running an initial split to 1-minute long chunks, some consideration needs to be made for when this pipeline is processed in parallel, where it is advantageous to start allocating files to machines to process as quickly as possible, and to give them shorter files such that work can be distributed more evenly. As such, this initial split length is used as an input parameter to test the distributed system to find an efficient configuration.

#### Silence removal

As discussed above, it is highly advantageous to remove audio before execution the MMSE STSA filter because of its long execution time. Audio containing heavy rain is already removed, but even more audio can be removed by detecting audio that does not contain any bird sound of interest. Because of this, we introduce a basic silence removal approach to the processing pipeline. This approach uses a simple threshold. The choice of threshold is derived next, based on one of two acoustic indices taken from Bedoya et al. [13]: Power Spectral Density (PSD), and Signal to Noise Ratio (SNR). Execution time testing shows that this silence detection approach takes a very short time relative to other processes, taking approximately 10 seconds to process 2 hours (1.2 GB) of audio, regardless of the split length.

Silence detection testing is now added to subsequent tests used in evaluating the processing pipeline.

#### Effect of the MMSE STSA filter on noise reduction

The MMSE STSA filter [14] is a process within the processing pipeline that reduces stationary background noise, hence, making signals clearer. However, this process is time consuming, as shown in Fig 1, so processes should only be applied after the MMSE STSA filter if they show significant improvement in detection accuracy, particularly if these processes remove audio, as removed audio does not need to be processed further. Here, we test the accuracy of rain, cicada, and silence filters before and after applying the MMSE STSA filter to determine where they belong in the pipeline, relative to the MMSE STSA filter.

We first evaluate the accuracy of rain and cicada detection when the MMSE STSA filter is applied. For this test, acoustic indices were calculated for raw audio, and audio processed by the MMSE STSA filter (although a 1 kHz high-pass filter was used for each set). The audio in each set was otherwise identical outside of processing.

The classification accuracies of each set are given in Table 2. This clearly shows that the MMSE STSA filter does not improve accuracy, and actually reduces it for rain detection. This is likely because rain has stationary and non-stationary components (i.e. raindrops distant from the sensor make a constant background noise, whereas closer raindrops are clearly audible and distinguishable). As such, the MMSE STSA reduces some, but not all of the noise sources, making them more difficult to detect.

**Table 2:** Comparison of detection accuracy depending on use of MMSE STSA filter.

Filter	Cicada Accuracy	Rain Accuracy
Raw	99.3%	96.9%
MMSE STSA	99.1%	92.9%

**Fig 3:**
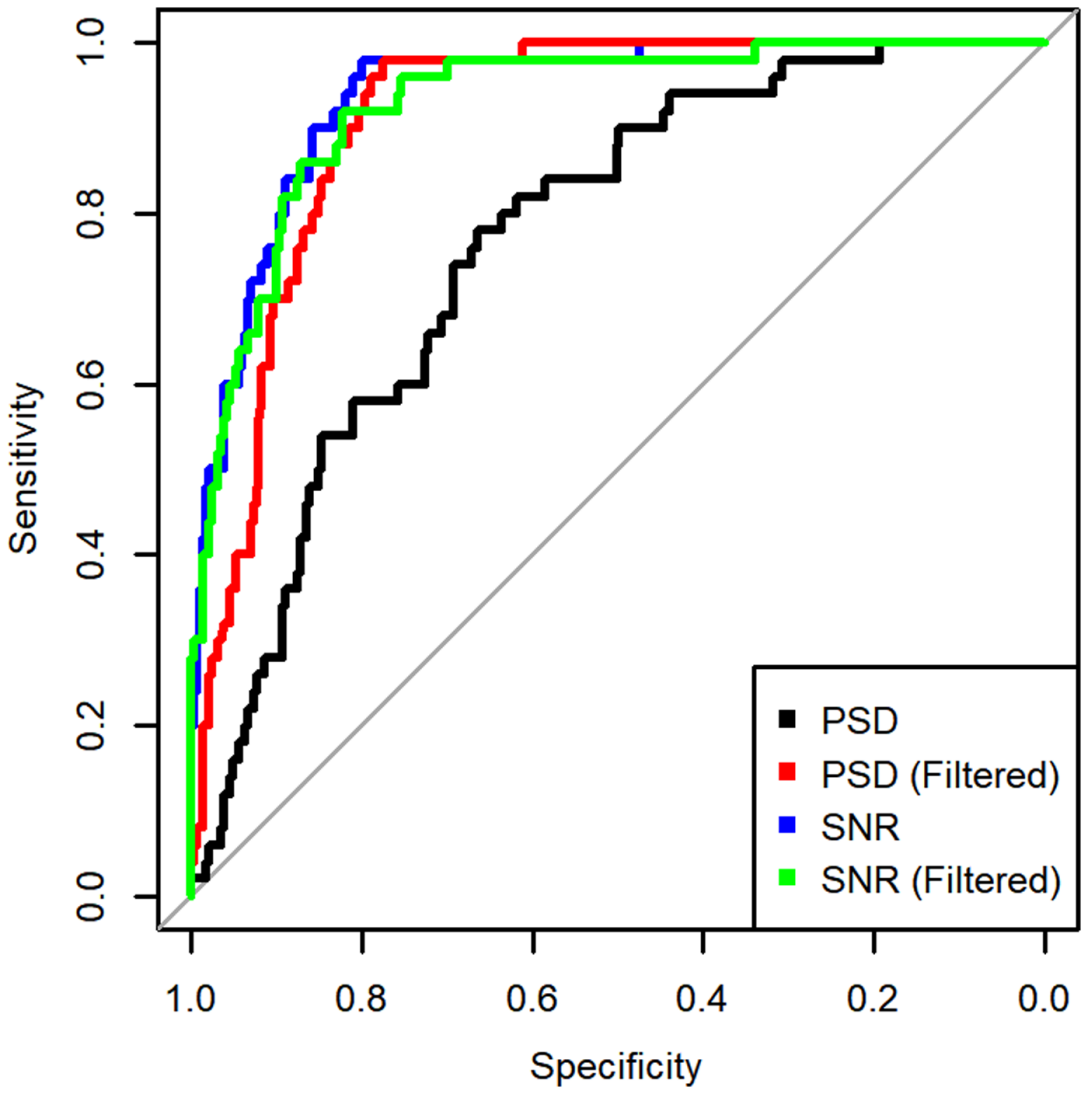
ROC curve for classifying silence.

For silence detection, thresholds using two different measures are considered: power spectral density and signal to noise ratio (SNR). These are applied to files with and without the MMSE STSA filter to evaluate accuracy. Because only one measure is used, an ROC curve (Fig 3) is employed to visualise the accuracy of the thresholds as they are increased, in terms of the sensitivity and selectivity. The Area Under the Curve (AUC) is taken for each threshold and recording set, shown in Table 3.

**Table 3:** Area Under the Curve (AUC) results for silence removal, with 95% Confidence Intervals (CI) for raw and MMSE STSA filtered audio using Power Spectral Density (PSD) and Signal to Noise Ratio (SNR) thresholds.

Audio Source	Index	AUC	95% CI
Raw	PSD	0.768	0.745–0.831
Raw	SNR	0.939	0.910–0.969
Filtered	PSD	0.913	0.8818–0.944
Filtered	SNR	0.929	0.894–0.964

The results of this show that, if using the Power Spectral Density measure, the MMSE STSA filter would be necessary to obtain good results. However, the SNR measure performs similarly well regardless of the use of the MMSE STSA filter. Because of the time cost of using the MMSE STSA filter, it is more efficient to execute silence detection based on SNR prior to executing the MMSE STSA filter.

#### Effect of split length on noise reduction

This section examines if detection approaches are dependent on split lengths. To do this, the accuracy of each detector (silence, rain, and cicada chorus) is tested on 30 minutes of audio composed by randomly selected 1-minute chunks spread over four days of original recordings. These chunks are then split into 5, 10, 15, 20, and 30 second chunks (these divide evenly into 60 seconds). These are listened to and manually labeled as rain, cicada, or silence, to a resolution of 5 seconds. Each detection approach is then tested for each split length. Manual labelling is performed on audio filtered by the MMSE STSA algorithm, even though automatic methods work with raw audio. This gives better accuracy for manual labelling, particularly for detecting silence, because very quiet calls become clearer.

Accuracy is evaluated for each split length to a precision of 5 seconds, despite the fact that these approaches do not have this level of precision for longer split lengths. For example, given a 10-second long chunk, if there is silence in the first 5 seconds, but a sound in the following 5 seconds, and that chunk is labelled as silence by the system, this is interpreted as one true positive and one false positive result, even though only one file was classified.

In practice, the silence classifier labels some rain as silence. This makes intuitive sense, given it is using an estimated signal to noise ratio (SNR) threshold, which is a measure of peak volume to average volume. If the average volume is very loud then the SNR will be low, even if the peak volume is also loud (compared to times when it is not raining). Despite technically being a false positive, this is not a significant issue, because rain is removed by the rain filter anyway. However, this creates a complication, because some rain samples contain audible rain drops, which results in files with a high signal to noise ratio. Consequently, because the silence filter detects some, but not all rain samples as containing silence, samples manually classified as containing rain were removed from the silence classification test.

For all figures in this section, the number of true positives, false positives, and false negatives are shown. True negatives are excluded from these figures as the number of true negatives is much greater than the others in every case, which makes visual comparison more difficult.

**Fig 4:**
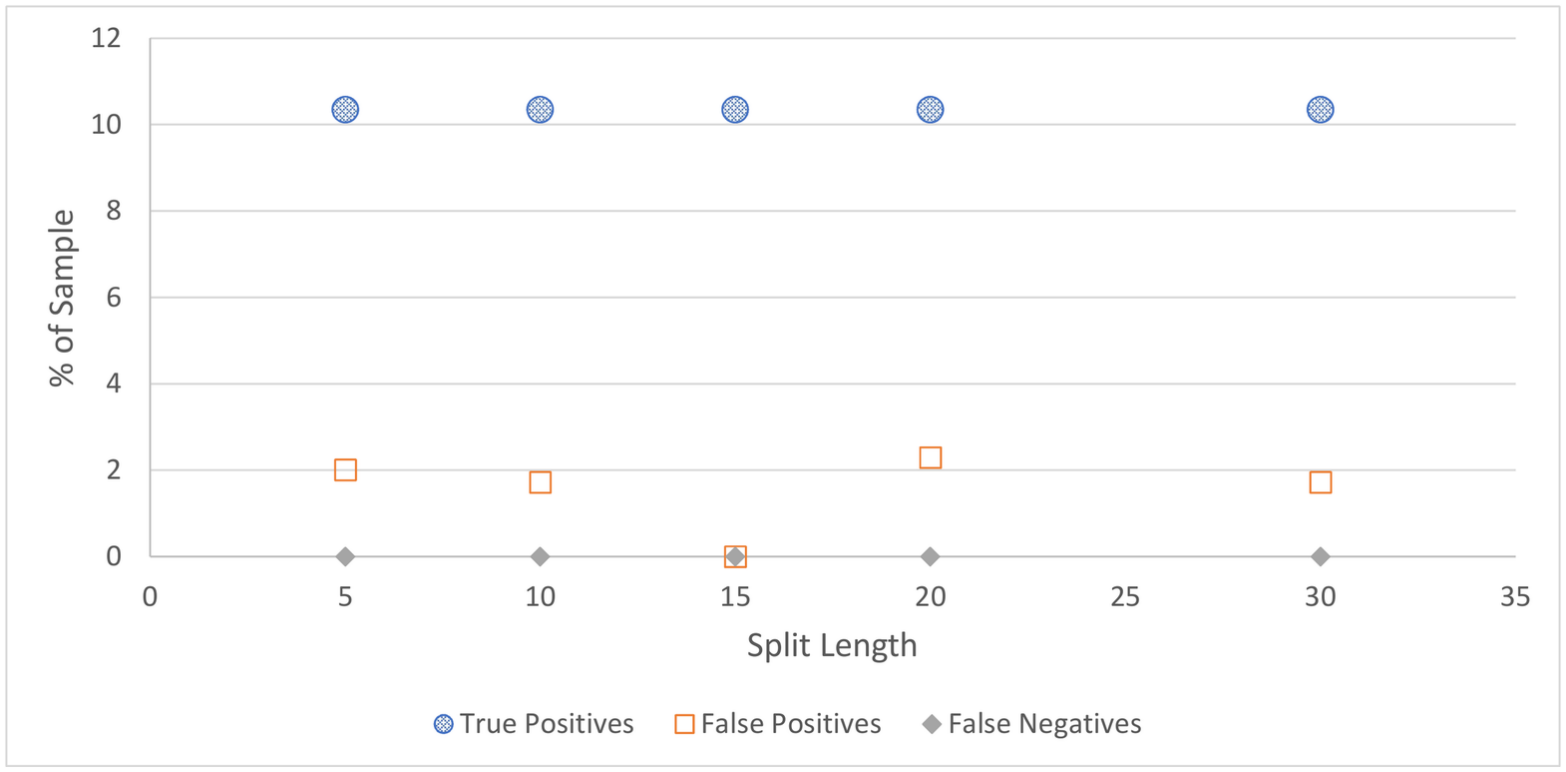
Results of cicada classification test.

**Fig 5:**
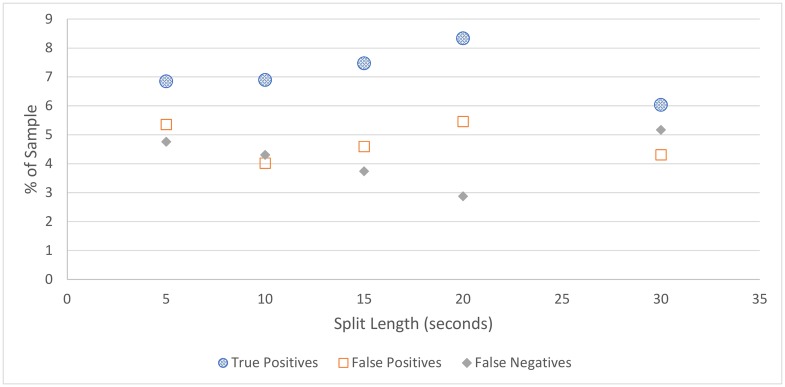
Amount of audio detected as rain in a sample as it varies with split length.

**Fig 6:**
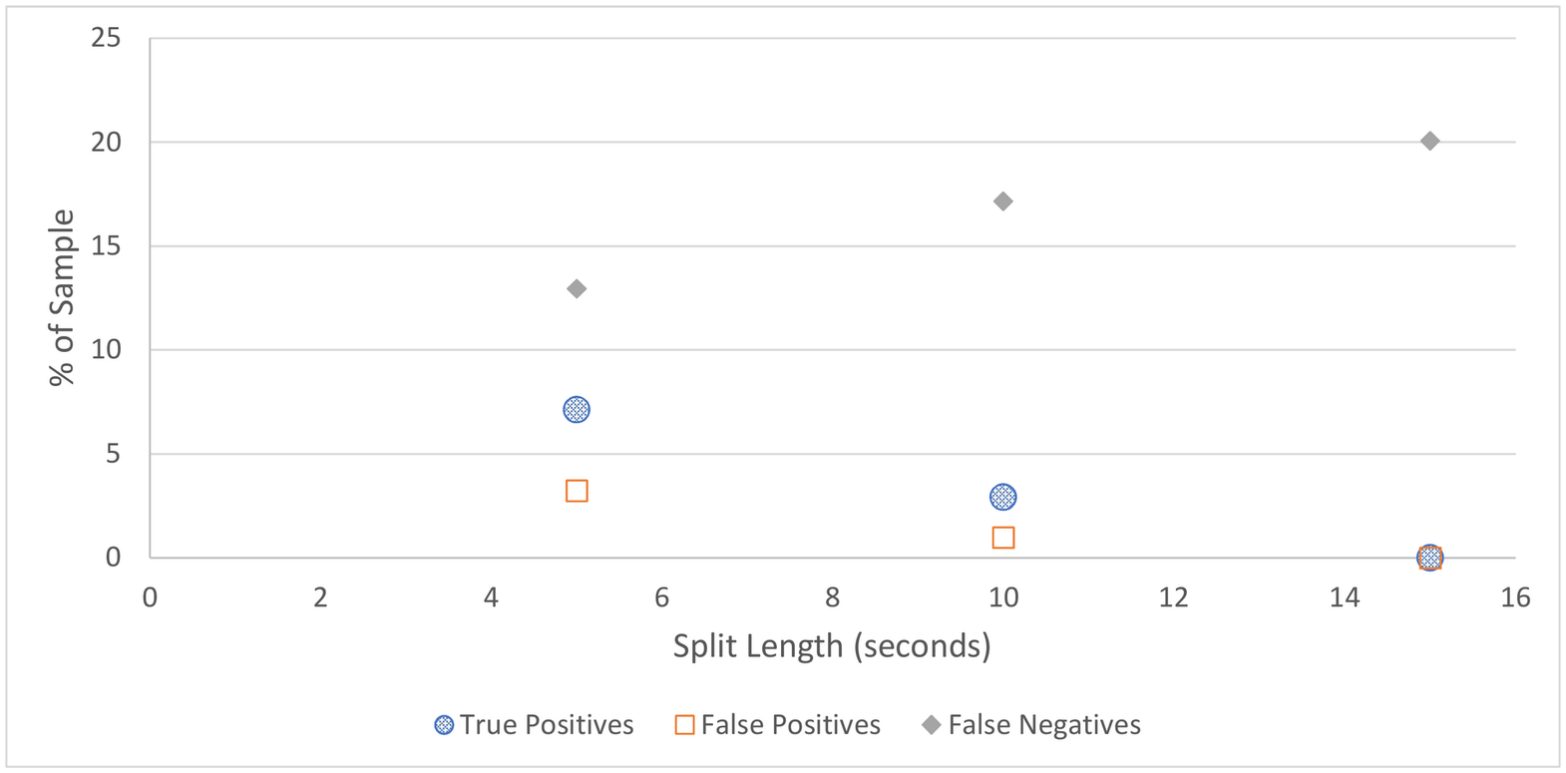
Silence detection accuracy for the higher of the two thresholds tested.

**Fig 7:**
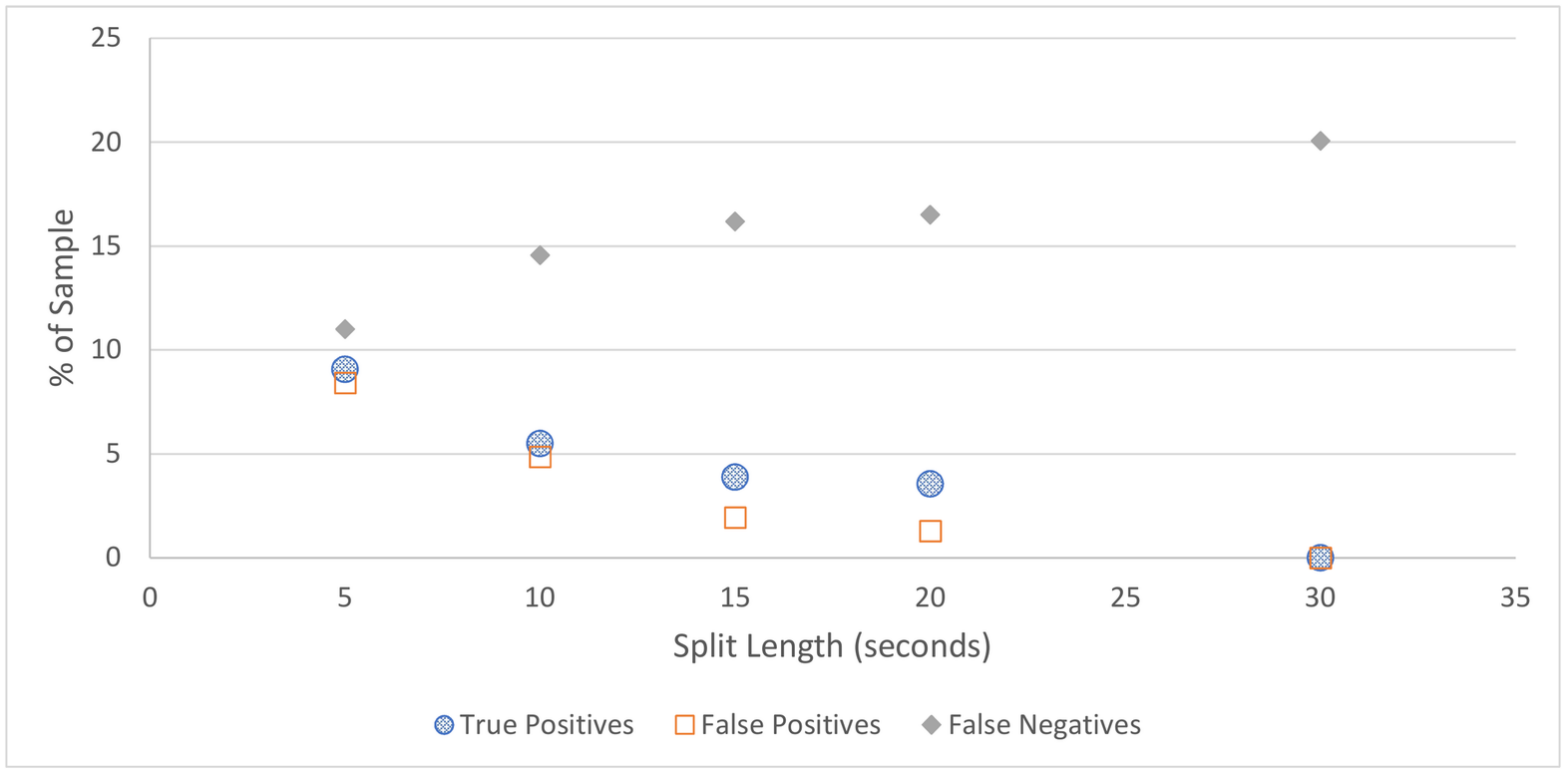
Silence detection accuracy for the lower of the two thresholds tested. All split lengths above 15 seconds detect no silence.

- **Cicadas:** The cicada detection results, depicted in Fig 4 and Table 4, shows that cicada detection works well for all split lengths, detecting all cicada choruses in the sample, with a small number of false positives. The best performing split length is 15 seconds, which contained no false positives, although this strong result could be partially due to chance.
- **Rain:** Similar to cicada detection, the amount of rain detected does not vary much depending on split length, as shown in Fig 5 and Table 5. Somewhat surprisingly, rain detection is slightly more sensitive, and more accurate, for longer split lengths, at least up to 30 seconds, at which point a steep drop-off occurs. This is likely because rain tends to occur over a long duration, and patterns that can be used to detect rain are clearer over longer time periods.
  In practice, the accuracy of rain detection is not as poor as this evaluation suggests. When manually labelling the data, only rain considered intense enough to drown out any bird signal was classified as rain, although the rain classifier classifies some lighter rain without significant bird sound as containing rain. While these are labelled as false positives, many of these would be (validly) removed by the silence detector anyway.
- **Silence:** Figs 6 and 7, and Table 6, show the accuracy of the silence detector at different signal to noise ratio thresholds. Unlike rain and cicada detection, split length has a significant effect on the sensitivity of silence detection. This is because silence is much more likely to occur over shorter durations.
  Overall, the silence detector performs somewhat poorly, producing about as many false positives as true positives on more aggressive settings, and failing to detect many instances of silence on all settings, with worsening performance for longer split lengths and lower thresholds. This indicates a better approach is needed for removing silence overall, which will be the subject of future work. For the present investigation a less sensitive threshold is selected, as this is more accurate overall and retains more samples containing bird sound, which is more important than any efficiency gained from removing silence, as these can be dropped at a later point. As such, the 5-second sample with the lower threshold is considered the best setting for our filter, which does remove over one third of silence, while classifying relatively few false positives. Though using 5 second splits means that the MMSE STSA filter takes longer to execute (see Fig 1), the effect of removing silence will have a greater effect on reducing execution time overall.
  It is notable that, while the silence detector does produce many false positives, the false positives contain quiet bird calls, not significantly louder than the background noise. Even after applying the MMSE STSA filter, noise still masks these faint calls, making them poor candidates for automated species identification. In our testing, the silence filter never removed any audio with very clear bird calls.

**Table 4:** Cicada detection accuracy.

Split Length	True Pos.	False Pos.	False Neg.	True Neg.	Accuracy
5	10.3%	2.0%	0.0%	87.6%	98.0%
10	10.3%	1.7%	0.0%	87.9%	98.3%
15	10.3%	1.7%	0.0%	89.7%	100.0%
20	10.3%	2.3%	0.0%	87.4%	97.7%
30	10.3%	1.7%	0.0%	87.9%	98.3%

**Table 5:** Rain detection accuracy.

Split Length	True Pos.	False Pos.	False Neg.	True Neg.	Accuracy
5	6.8%	5.4%	4.8%	83.0%	89.9%
10	6.9%	4.0%	4.3%	84.7%	91.7%
15	7.5%	4.6%	3.7%	84.2%	91.7%
20	8.3%	5.5%	2.9%	83.3%	91.7%
30	6.0%	4.3%	5.2%	84.5%	90.5%

**Table 6:** Silence detection accuracy.

Split Length	True Pos.	False Pos.	False Neg.	True Neg.	Accuracy
*SNR threshold* = *0.25*					
5	9.1%	8.4%	11.0%	71.5%	80.6%
10	5.5%	4.9%	14.5%	78.0%	80.5%
15	3.9%	1.9%	16.2%	78.0%	81.9%
20	3.6%	1.3%	16.5%	78.6%	82.2%
30	0.0%	0.0%	20.7%	79.9%	79.9%
*SNR threshold* = *0.2*					
5	7.2%	3.3%	12.9%	79.9%	83.8%
10	2.9%	1.0%	17.2%	78.9%	80.0%
15	0.0%	0.0%	20.1%	79.9%	79.9%

#### Work distribution between the master and slaves

Based on our investigation of individual processes, we perform downsampling and high-pass filtering alongside splitting using the master process. The time taken to perform these steps is small compared to the overall processing time of the pipeline, so executing these steps serially does not increase processing time. Downsampling reduces file sizes which means that less time and bandwidth is used in sending files to slaves. High-pass filtering is performed on the master process because it utilises long split lengths. By doing this on the master process, files can be split into shorter chunks for distribution.

### Final pipeline

**Fig 8:**
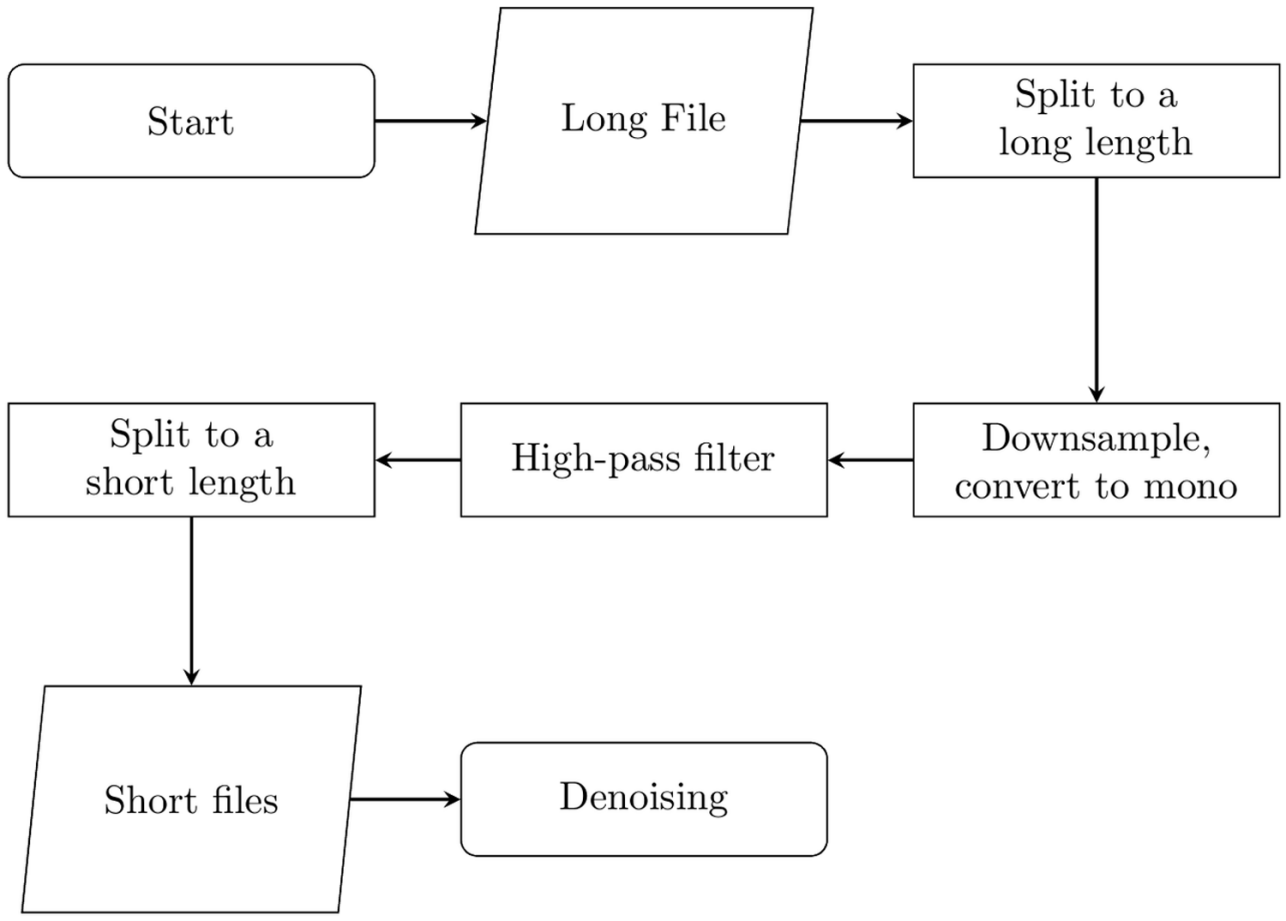
Early steps of the processing pipeline, processed by the master. The “long length” and “short length” are determined in subsequent tests.

**Fig 9:**
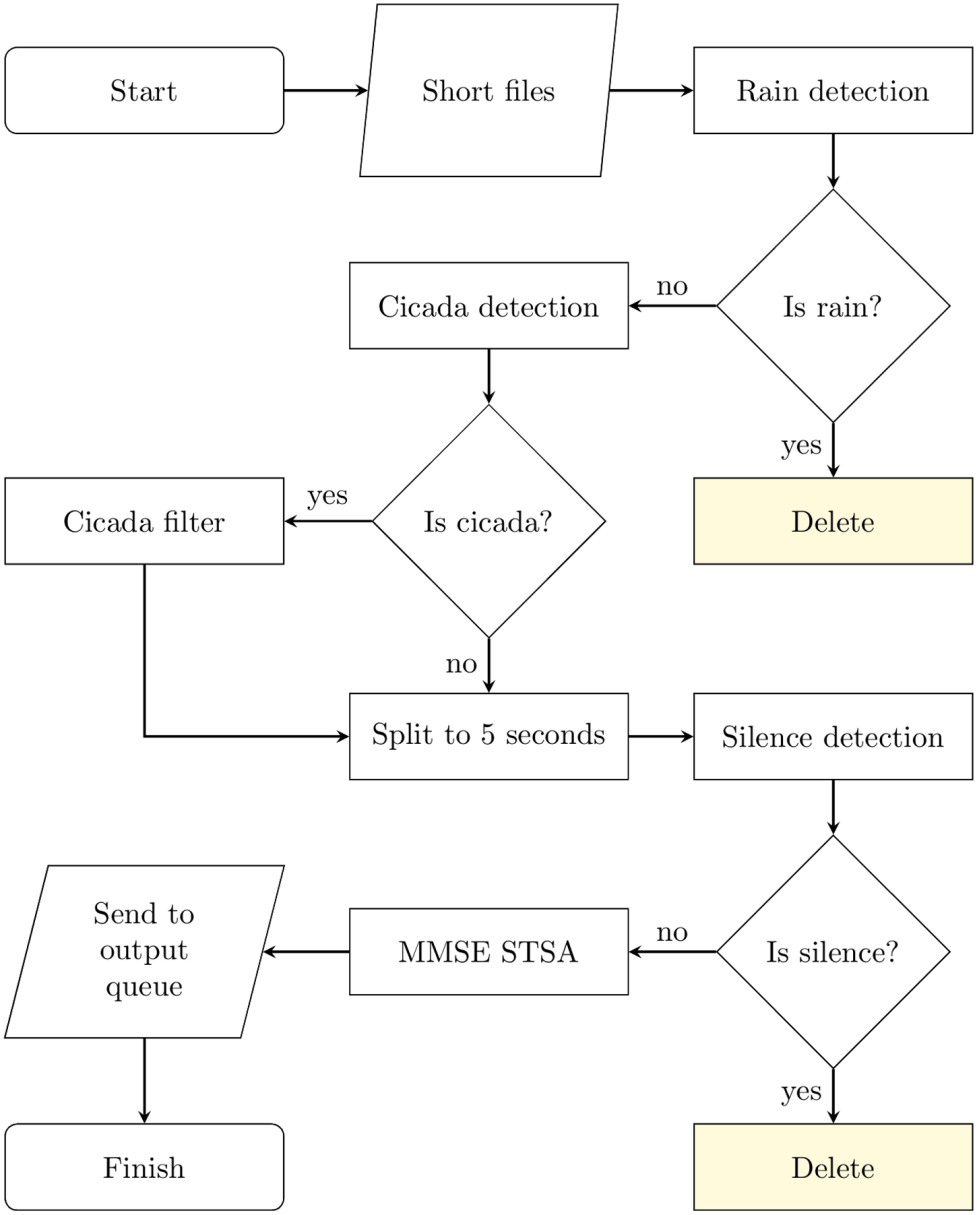
Denoising steps of the processing pipeline, processed by the slaves.

Based on the above findings and evaluation results from the previous sections, the final pipeline for preprocessing bioacoustics recording based on denoising filters is given in Algorithm 1 and summarised in Figs 8 and 9.

Files are first split to break up processing into smaller steps which can be parallelised. Compression processes are then applied to reduce execution time of all other processes. High-pass filtering is applied, removing any noise below 1 kHz and improving detection mechanisms. This also works better with longer split lengths, so applying earlier improves execution times. Then rain and cicada detection are executed, with rain detection executing earlier because it may eliminate audio from further processing. Files are then split to 5 seconds, before silence detection is performed. Finally, the MMSE STSA filter is executed. Placing this at the end reduces execution time because any files removed by other processes do not need to undergo MMSE STSA filtering, which has the longest execution time of any individual process.

Importantly, any file removed in earlier processes does not need to complete the pipeline, saving significant execution time. Hence, silence and rain detection steps significantly improve execution times, while resulting in higher quality output because useless chunks are discarded. In particular, skipping the MMSE STSA step removes the majority of processing time of any given file.

### Identifying best settings for efficient workflow distribution

Now that the pipeline has been constructed, the next step is to integrate it into a distributed system, and determine the best configuration for this system. A large number of configurations are examined to find which set produces the fastest execution. In particular, the split length, the split length before applying the high-pass filter, referred to here as the *long split length*, the maximum queue size of slaves’ central threads, and the interval between slaves sending results are considered. These tests are carried out using 4 virtual machines with 4 cores each and 16 GB of RAM. These machines are hosted in the Nectar Cloud, which is a cloud platform used by Australian and New Zealand universities.

**Algorithm 1** Processing Pipeline

Split an audio file into “long” chunks (the best length for these will be tested subsequently)

**for all** “long” chunks **do**

 Downsample audio to 22.05 kHz and convert to mono

 Apply a 1kHz high pass filter

 Split audio into “short” chunks (the best length for these will be tested later)

 **for all** “short” chunks **do**

  Apply an STFT

  **if** chunk contaminated by rain **then**

   Delete chunk

  **else**

   **if** chunk contains cicada chorus **then**

    Identify chorus frequency range

    Apply Sinc Filter to elimiate cicada chrous

   **end if**

   Split chunk to 5 second long sub-chunks

   **for all** sub-chunks **do**

    **if** sub-chunk is silent **then**

     Delete sub-chunk

    **else**

     Apply MMSE STSA filter

    **end if**

   **end for**

  **end if**

 **end for**

**end for**

Initially, we conduct ad hoc tests using a large number of different parameter sets to reduce the number of configurations to undergo more thorough testing to a more manageable level. In these tests, each set is only tested once. From this ad hoc testing, parameter ranges are set to evaluate 90 configurations in more depth. Each test is conducted five times each with the same two hours of audio used in earlier tests being processed each time. Of these, 10 configurations with the lowest average execution time are shown in Table 7.

**Table 7:** Ten best configurations identified in distribution testing.

Split length (s)	Long split length (s)	Max queue size	Time per send (s)	Average execution time (s)	Std. dev. (s)
10	120	7	2	72.55	1.14
20	60	5	2	72.74	0.90
10	60	5	2	72.75	0.56
5	120	7	3	72.76	1.13
30	60	3	2	72.95	0.42
10	120	5	3	72.95	0.45
15	60	5	3	73.14	0.70
5	60	7	4	73.14	1.41
10	60	7	2	73.15	1.00
20	60	3	2	73.15	1.58

A key insight from these results is that there is little difference in performance between the best configurations, with the top 10 being separated by 0.6 seconds over 2 hours (1.2 GB) of audio (0.8% of the fastest time) and well within the standard deviation of all the top 10. The fastest configuration equivalently processes audio at a rate of 16.4 ± 0.3MB*s*^−1^, or 99.2 ± 1.6 seconds of audio processed per second of real time (error given by the standard deviation). The only poor combination is to have a split length of 5 and maximum slave queue size of 3, and any combination of other settings. These configurations are about 25 seconds slower on average than any other configurations. The top 84 configurations (i.e. all configurations except the known bad ones) are separated by 8.03 seconds (this becomes 2.81 seconds for the top 50), which is statistically significant, so there is a small time efficiency advantage from thoroughly testing configurations as opposed to selecting one at random.

This indicates that configurations can be selected for accuracy, without significant loss of efficiency. Because splitting into 15 second chunks is the most accurate approach for removing rain and cicada sounds, this is taken to be the split length in further testing. This gets split into 5 second chunks for silence detection at a later point of the pipeline.

## Performance evaluation of preprocessing pipeline

Given the determined efficient order of execution for the preprocessing steps and determined how to distribute the resulting pipeline efficiently, we evaluate the system’s performance for preprocessing high volume data. We perform multiple tests, comparing execution times, load balancing, and resource usage over different numbers of resources, some of which have different levels of processing power.

### Execution time and scalability testing and analysis

We first evaluate the system’s ability to scale given differing amounts of processing power. The system is tested using two hours of audio known to contain bird sound, rain, cicada choruses, and silence with varying numbers of machines. The test is run four times for each case, and the average execution time recorded. The 1-core execution test uses a process specifically written for sequential execution, while the others uses the distributed system. The CPU count includes the master and slave nodes. Because the master node does not require a large amount of resources, a slave node is also executed on the same machine as the master. Each instance tested contained 4 cores and 16 GB RAM, though most of this RAM is not used by the system. The 2-core case was tested using a single 2-core instance running a master and a slave process.

**Fig 10:**
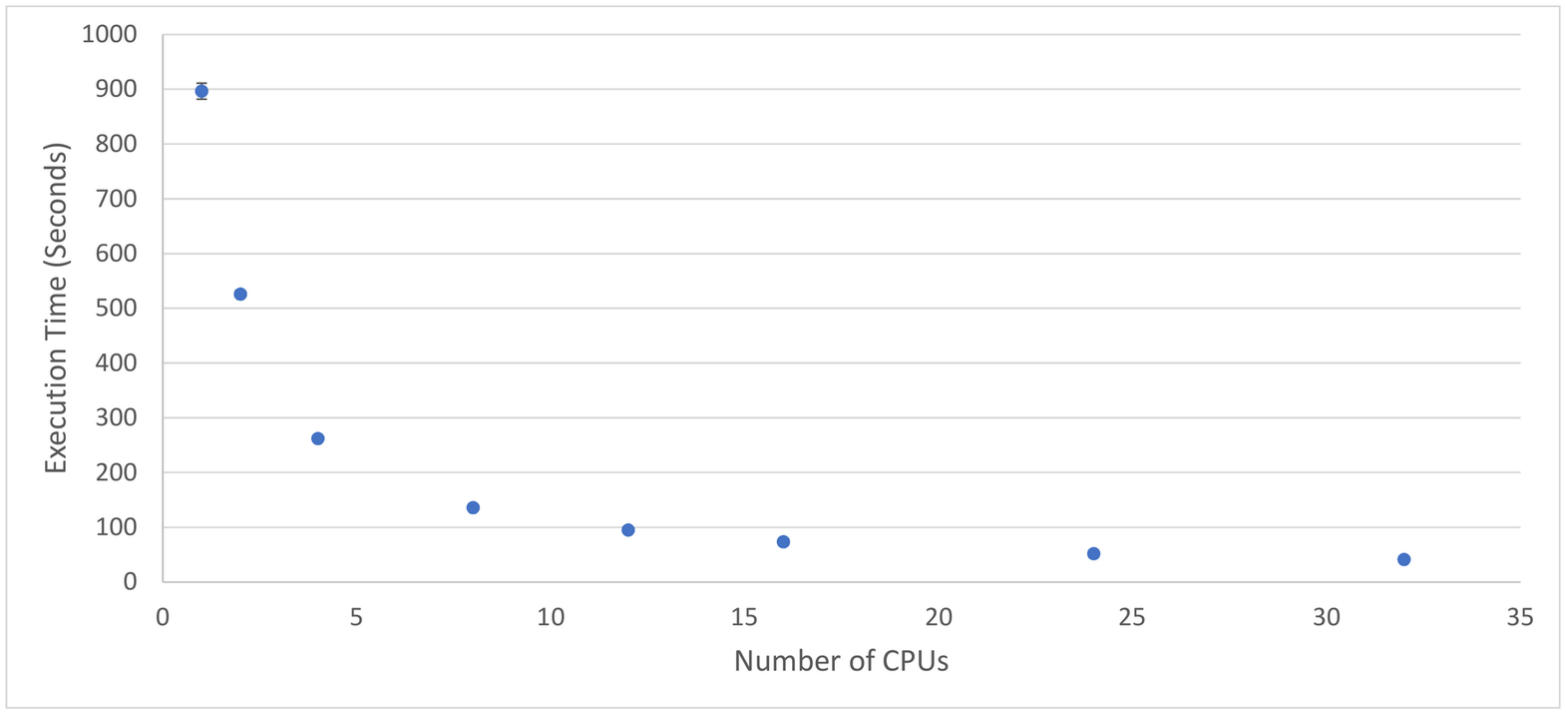
Average execution time of the system given a number of cores. The master and each slave have 4 cores, so 16 cores uses 4 virtual machines. Standard deviations are too small (4.9 seconds at most) for most error bars to be visible.

**Fig 11:**
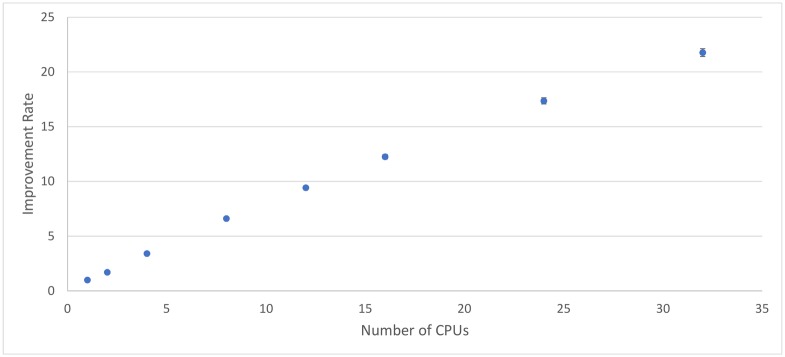
Rate of improvement in execution time per number of cores. This is given by Execution Time of 1 core/Execution time of *x* cores.

Fig 10 shows the average execution time for the number machines used. Fig 11 presents the improvement in the execution time over 1 core by measuring how many times faster execution is compared to the sequential (1-core) case. Table 8 gives a numerical summary of results.

**Table 8:** Execution times for processing 2 hours of audio for different distributed system configurations. The master and each slave have 4 cores, so 16 cores uses 4 virtual machines. Improvement rate is given by execution time of 1 core/Execution time of *x* cores.

# Machines	#Cores	Execution Time	Improvement Rate
1	1	896.6 ± 14.5	1×
1	2	526.0 ± 6.1	1.70 ± 0.03×
1	4	262.3 ± 1.4	3.42 ± 0.06×
2	8	135.6 ± 2.0	6.61 ± 0.11×
3	12	95.2 ± 1.9	9.42 ± 0.15×
4	16	73.2 ± 1.9	12.25 ± 0.20×
6	24	51.7 ± 0.6	17.35 ± 0.94×
8	32	41.2 ± 0.9	21.76 ± 0.35×

The first investigation considers the execution time of the pipeline in serial (1 core). Note that [naively] adding the execution times for all individual processes given 15 second splits (see Table 1) suggests a likely overall processing time of 1251.2 plus/minus 3.7 seconds. However, with our processing pipeline, which removes some audio earlier in processing, negating the need to perform all processing steps, the same processing is achieved in 896.6 ± 14.5 seconds. This gives a 1.4 times improvement, simply by considering the order of execution for the pipeline and avoiding redundant computations.

Fig 11 shows that the system is indeed scaling almost linearly, with significant speed boosts from using extra processors. The improvement rate does begin to slightly diverge from perfect linearity when high numbers of cores are used, but even a 32-core distributed system still shows significant performance increases over a 24-core system. There is also a slight statistical anomaly where the 2-core system does not improve as much over the sequential 1-core system as might be expected. This is likely because of the extra overhead involved in using the distributed system over the sequential system. However, this extra overhead does not seem to prevent the system from being linearly scalable.

A further test is conducted using smaller machines which, when combined, give a similar power level to large machines. The configurations compared are as follows:

1. One 4-core, 16 GB RAM master, one 4-core, 16 GB RAM slave
2. One 4-core, 16 GB RAM master, two 2-core, 6 GB RAM slaves
3. One 4-core, 16 GB RAM master, four 1-core, 4 GB RAM slaves

The master also runs a slave instance in all cases, to make a fairer comparison with the previous tests. This also has the effect of testing system performance where different sizes of virtual machines are operating at the same time, as the master virtual machine runs a slave with 4 cores in all cases, albeit while competing for resources with the master thread.

**Fig 12:**
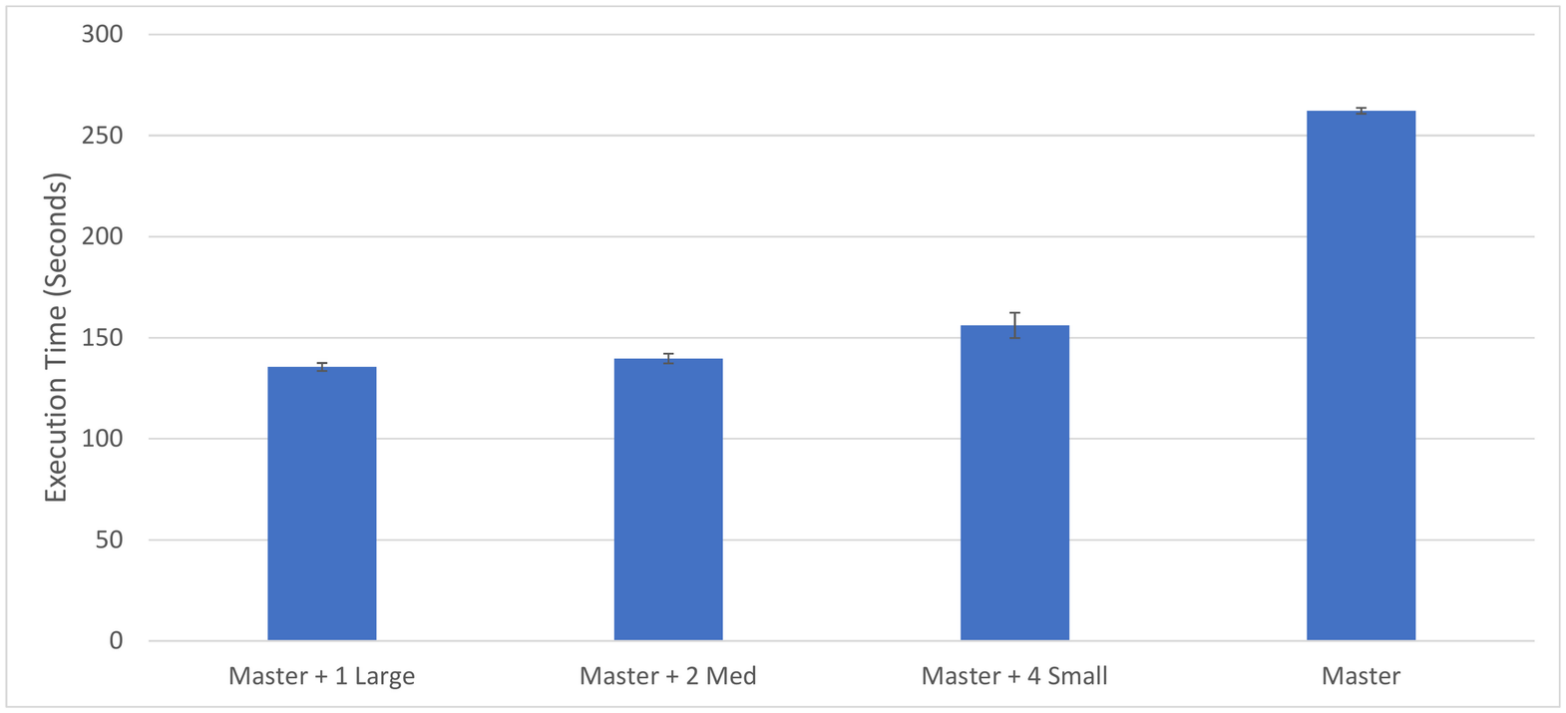
Execution time comparison between using more smaller machines and using fewer larger machines. The master on its own is also shown for comparison.

The results shown in Fig 12 indicate that the system works as well with the master and two 2-core slaves compared to the master and one 4-core slave, and slightly worse when four 1-core slaves are used. The slower execution time when using 1-core machines could be due to extra overhead caused by the use of the centralised slave thread. This use of the central slave thread (which can be further broken down into six small threads) results in excessive overhead with smaller machines, while with larger machines reducing the amount of communication to the master and waiting times in processing files become advantageous. It could also be due to an inappropriate queue size being used for smaller machines, leading to imbalances in workload during later stages of execution. The system is developed for larger machines, so it makes intuitive sense that they would compute faster. Overall, the system is capable of performing efficiently with virtual machines of any size, although slightly less efficiently when 1-core machines are used. It also shows that the system can maintain efficiency when machines of different sizes are processing at once, because the master is running a slave thread with 4 available cores in all tests.

### Load balancing testing and analysis

We also conduct an analysis of load balancing at the same time as the scalability tests. This measures how many files are going to each of the slaves. Because all the slave machines have identical specifications, the file distribution should be even in an ideal case, apart from one slave which will have a lower number of files because it is sharing resources with the master process.

As shown in Table 9, while work is not distributed entirely evenly for all cases (particularly when four 4-core machines are used), it is very close to being so.

**Table 9:** Load balance distribution across different numbers of machines. The maximum and minimum load refer to the average load of VMs with the largest and smallest loads (in terms of percentage of files processed) over five trials. The *p*-value is derived from a single-factor ANOVA test. *p* ≤ 0.05 indicates, with 95% confidence, that processing loads are not equal.

# Machines	Max Load	Min Load	*p*-value (ANOVA)
2	51.7±2.0%	48.3±2.0%	0.026
3	34.1±2.6%	32.7±1.9%	0.54
4	26.2±0.7%	23.9±0.5%	<0.001

Table 10 demonstrates that the system is capable of balancing workload where the machines being used are of unequal power. This data are taken from earlier tests where the master, with 4 cores, is running a slave process simultaneously and less powerful machines are also running slave processes. Here, the machine running the master process correctly allocates more files to itself compared to what it allocates to each of the slaves, proportional to the differences in computing power, though more computation is done by the machines with fewer cores overall, because the 4-core machine is also executing the master process.

**Table 10:** Load balance distribution across different numbers of machines that have differing numbers of CPU cores. In each test, one machine has 4 cores and executes the master process alongside a slave process, and the smaller machines run slave processes. The maximum and minimum load refer to the average load of VMs with the largest and smallest loads (in terms of % of files processed) over five trials. The *p*-value is derived from a single-factor ANOVA test. *p* <= 0.05 indicates, with 95% confidence, that processing loads are not equal.

# Machines	# Cores	4-Core Load	Max Load	Min Load	*p*-value
2	2	46.8±1.2%	27.2±1.9%	26.1±1.6%	0.34
4	1	46.4±0.9%	14.2±1.1%	12.3±0.9%	0.10

### Resource usage test and analysis

A test is conducted to see how efficiently the system is using resources. This is done by processing two hours of audio with four slaves, and sampling the CPU and RAM usage approximately every 8 seconds. This sampling is done using a shell script running in parallel to Java execution, although some data regarding timing is sent to the debugging logs to help synchronise the timings between slaves. While accuracy of the times is imperfect, it should be accurate to within 3 seconds.

**Fig 13:**
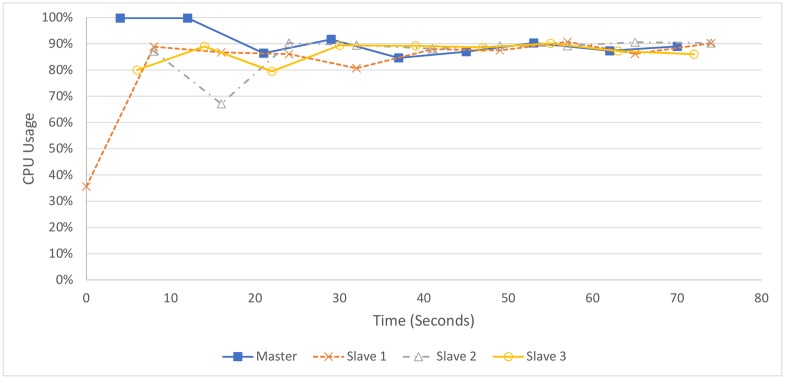
CPU usage over four 4-core machines processing 2 hours (1.2 GB) of audio.

Fig 13 shows that CPU usage remains at about 90% for most of the processing of the two hours of audio. There does appear to be a slight drop below this number at the start of processing, presumably due to the master still performing early processing and not having files to send. Overall, assuming the overhead is not significant to CPU usage, it would be difficult to significantly improve upon the current pipeline without changing the pipeline itself. Note that the master is also running as a slave, and the master CPU usage relates to the usage by the slave and master processes running on that machine.

**Fig 14:**
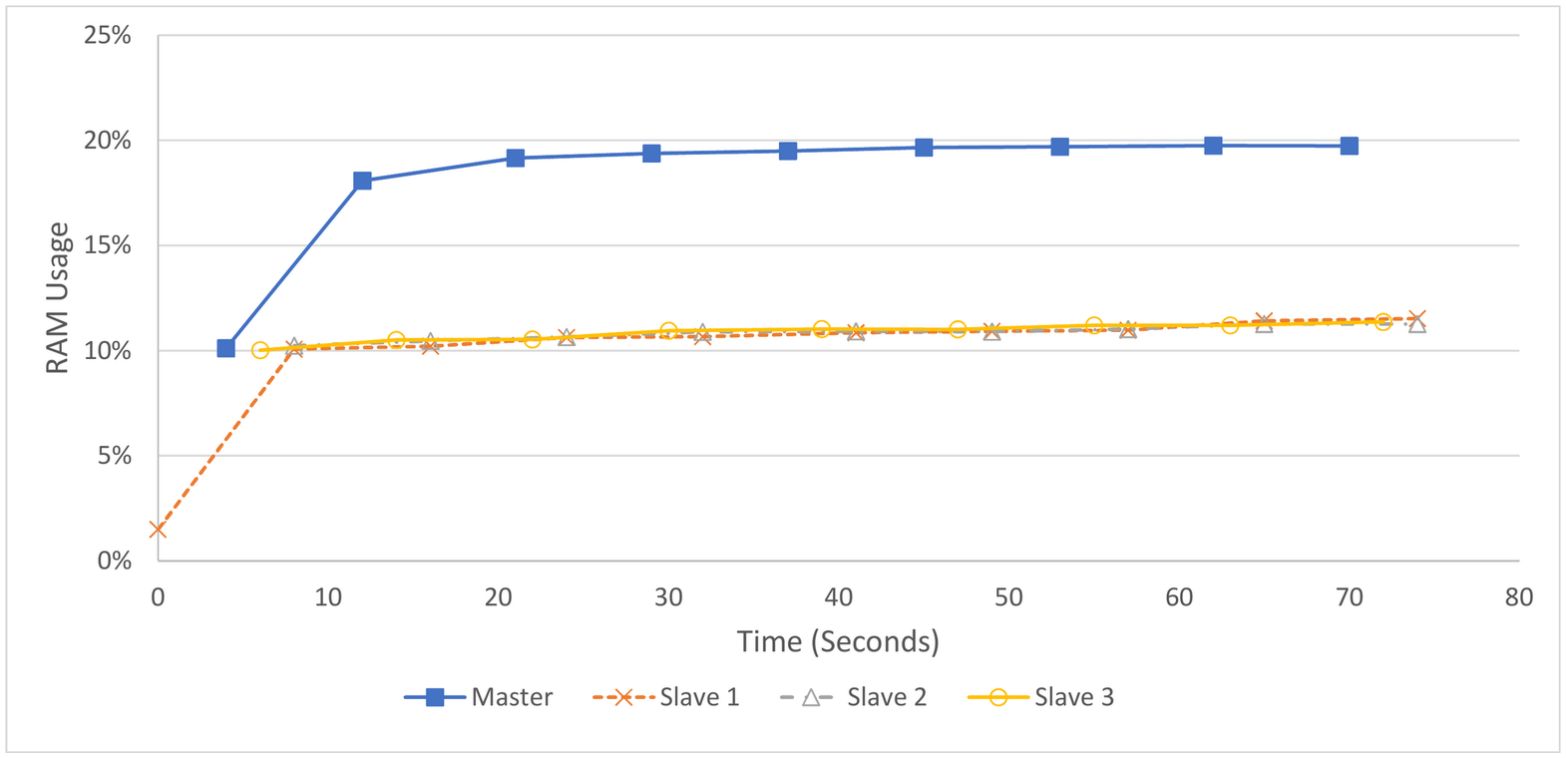
RAM usage over four 4 core machines processing 2 hours (1.2 GB) of audio.

Fig 14 shows that the three slaves utilise around 11% of the machines’ 16 GB of available RAM, remaining constant after the first 10 seconds. The master uses more RAM, presumably due to holding information about slave sockets and data streams, as well as information about files, relating to whether they have been sent and which slave is processing them, in addition to running a slave process.

RAM is underutilised overall. The system relies heavily on file writes and file reads using data storage, which results in low RAM utilisation. Keeping more data in RAM could result in faster memory access and in turn, faster processing. However, as CPU usage is already fairly high, hard disk reading and writing does not seem to be a significant bottleneck in processing these audio files. Nonetheless, this is a potential area for performance improvements in future work.

### Comparison with similar approaches

Dugan et al. [22] focus their cloud infrastructure on completing two tasks: auto detection and noise analysis. In each of these, a process manager divides work into *M* nodes which each independently work on their own tasks. Their sensor data is multiplexed in the data files (i.e. data from multiple sensors are shared in the one place), so data are divided by time, rather than by sensor. Recordings for the time period to be analysed are split into blocks equal to the number of processing nodes and each of these blocks are assigned a node. Nodes process independently, then return their output. Using this they found that, while speed improvements varied between the process being tested, the most improved process (classifier-based detection) was 6.57× faster for an 8-node server over a serial process, although another process (template-based detection) only improved by 3.33× over a serial process using an 8-node server running in parallel. A drawback to their approach is the use of a MATLAB package to handle distribution, which, while easier to develop, lacks low-level control over the data, and adds overhead. They have expanded this work with numerous publications, such as in a 2015 work [34] where they built an Acoustic Data-Mining Accelerator (ADA), which parallelises mapping and gathering operators in an otherwise sequential process.

Truskinger et al. [21] aim to extract acoustic indices to visualise their bioacoustics data. To do this, they distribute work by splitting audio into smaller chunks, similarly to Dugan et al. [22]. The research claims it is not feasible to process audio files any longer than two hours due to the high amounts of RAM required, so they use a specialised program called mp3splt to divide the audio into 1-minute long chunks. A master task creates a list of work items for work tasks to do. Each work task is given a different chunk of audio to analyse. The results of these tasks are aggregated by the master task. Through this parallelisation, the execution time of an analysis task involving the computation of spectral indices is improved by a modest 24.00× for a 5 instance, 32 thread (with 32 cores per instance) distributed cluster over a single threaded process. While certainly an improvement, the parallelisation appears inefficient as the improvement rate is much lower than the increase in resources. While discussion of the pipeline is not detailed in the paper, a possible reason for this low improvement rate is that there is a large serial component to the processing pipeline used and so the parallel processors are not fully utilised.

Thudumu et al. [25] have developed a scalable framework to process large amounts of bioacoustics data using Apache Spark Streaming [24] and the Hadoop Distributed File System (HDFS) [23] which utilises a master-slave model. The system parallelises the chunking of audio data and the generation of spectrograms. Parallelisation is handled by Hadoop and Spark. For a task involving splitting 1 GB of audio into 10 second chunks and generating spectrograms, the system showed a 4.50× improvement in execution time in a test with a 1 core master node and a 4 core slave node, but a weaker 7.50× improvement in execution time with a 1 core master and three 4 core slaves compared to a serial process, indicating the system is not as scalable as it could be. Using an equivalent number of processing resources, our system achieves a 9.98× improvement, with a much more computationally intensive processing pipeline.

## Conclusions and future directions

In this work, we addressed the problem of efficiently pre-processing high volume bioacoustics data. In particular, we investigated how to efficiently sequence pre-processing tasks, while also considering their effect on others. We also investigated how to distribute these pre-processing tasks among multiple machines. We did this by examining the processing time and accuracy of individual preprocessing tasks, and how these changed depending on the order in which tasks are processed and how the audio is split into smaller chunks. We then applied the resulting processing pipeline in a distributed architecture designed specifically for processing this pipeline. We utilised data parallelism to distribute processing work.

In testing individual components of the system, we found that the MMSE STSA filter consumes a very large amount of the execution time, meaning this should be executed as late as possible in the sequence. We also found that high-pass and cicada filtering using SoX consumes more time when more, shorter files are being processed compared to fewer, longer files, which gave rise to an efficiency improvement. From these findings, we were able to derive a processing pipeline that executes 1.40 times faster compared to manually executing all preprocessing tasks in order.

Upon applying this processing pipeline in a distributed system, we are able to achieve near-linear scalability, even when using 32 cores, which improves execution time by 21.76 times over serial processing. This compares favourably to existing research. It has also been found that the system balances load evenly between machines, and can proportionally distribute more files to more powerful machines. Cores on all machines are found to consistently utilise 90% of their available power, though RAM is underutilised.

While this work presents a strong basis for creating a fast, efficient, and scalable bird acoustic preprocessing pipeline, there is great potential for expansion in the future. Silence detection currently performs poorly and is limited in that it can only choose to keep or drop 5-second long chunks. This is not a large problem for the present investigation, as we are more concerned with combining existing preprocessing filters together to efficiently process data. However, if we wanted to improve the accuracy and utility of our pipeline, we could replace our relatively simplistic approach with one of many existing segmentation processes, which divide animal calls into syllables, often being insensitive to noise (e.g. [35, 36]). Additionally, the cicada filter operates under a big assumption that bird sound within the same frequency region is irrecoverable, which might not necessarily hold true. Potentially, a filter could by designed to preserve these bird sounds, or at least, testing could be done to investigate if this assumption is valid.

This processing pipeline is simple and generic enough such that additional noise reduction techniques could be added to the pipeline without difficulty. Adding additional processes to the pipeline would likely mean nothing more than inserting a new process in between two existing ones. However, the extra processes’ impact on the execution time and effectiveness of the existing pipeline would need to be examined to maintain high efficiency and effectiveness. Although this work focuses on the removal of noise from two sources, cicada choruses and rain, there are many other noise sources that could be targeted in the future.

## Supporting information

S1 File Source code for used for this paper can be found at https://sourceforge.net/projects/fast-bioacoustics-processing/. (ZIP)
